# Developing a Theory of Change for a Digital Youth Mental Health Service (Moderated Online Social Therapy): Mixed Methods Knowledge Synthesis Study

**DOI:** 10.2196/49846

**Published:** 2023-11-03

**Authors:** Shane Cross, Jennifer Nicholas, Shaminka Mangelsdorf, Lee Valentine, Simon Baker, Patrick McGorry, John Gleeson, Mario Alvarez-Jimenez

**Affiliations:** 1 Orygen Melbourne Australia; 2 Centre for Youth Mental Health University of Melbourne Melbourne Australia; 3 Healthy Brain and Mind Research Centre Australian Catholic University Melbourne Australia; 4 School of Behavioural and Health Sciences Australian Catholic University Melbourne Australia

**Keywords:** adolescence, adolescent, blended care, blended, co-design, development, digital health, digital intervention, digital mental health, framework, hybrid, mental health, model, platform, self-determination theory, service, services, theory of change, therapy, youth mental health, youth

## Abstract

**Background:**

Common challenges in the youth mental health system include low access, poor uptake, poor adherence, and limited overall effectiveness. Digital technologies offer promise, yet challenges in real-world integration and uptake persist. Moderated Online Social Therapy (MOST) aims to overcome these problems by integrating a comprehensive digital platform into existing youth mental health services. Theory of change (ToC) frameworks can help articulate how and why complex interventions work and what conditions are required for success.

**Objective:**

The objective of this study is to create a ToC for MOST to explain how it works, why it works, who benefits and how, and what conditions are required for its success.

**Methods:**

We used a multimethod approach to construct a ToC for MOST. The synthesis aimed to assess the real-world impact of MOST, a digital platform designed to enhance face-to-face youth mental health services, and to guide its iterative refinement. Data were gathered from 2 completed and 4 ongoing randomized controlled trials, 11 pilot studies, and over 1000 co-design sessions using MOST. Additionally, published qualitative findings from diverse clinical contexts and a review of related digital mental health literature were included. The study culminated in an updated ToC framework informed by expert feedback. The final ToC was produced in both narrative and table form and captured components common in program logic and ToC frameworks.

**Results:**

The MOST ToC captured several assumptions about digital mental health adoption, including factors such as the readiness of young people and service providers to embrace digital platforms. External considerations included high service demand and a potential lack of infrastructure to support integration. Young people and service providers face several challenges and pain points MOST seeks to address, such as limited accessibility, high demand, poor engagement, and a lack of personalized support. Self-determination theory, transdiagnostic psychological treatment approaches, and evidence-based implementation theories and their associated mechanisms are drawn upon to frame the intervention components that make up the platform. Platform usage data are captured and linked to short-, medium-, and long-term intended outcomes, such as reductions in mental health symptoms, improvements in functioning and quality of life, reductions in hospital visits, and reduced overall mental health care costs.

**Conclusions:**

The MOST ToC serves as a strategic framework for refining MOST over time. The creation of the ToC helped guide the development of therapeutic content personalization, user engagement enhancement, and clinician adoption through specialized implementation frameworks. While powerful, the ToC approach has its limitations, such as a lack of standardized methodology and the amount of resourcing required for its development. Nonetheless, it provides an invaluable roadmap for iterative development, evaluation, and scaling of MOST and offers a replicable model for other digital health interventions aiming for targeted, evidence-based impact.

## Introduction

### Overview

Poor youth mental health is a global problem. Mental illness is the leading cause of disability worldwide [1], and 75% of all mental disorders have their onset before the age of 25 years, severely disrupting the social, emotional, and vocational transition to adulthood [2], with life-long consequences [3]. While significant progress and investment have been made worldwide in developing a youth mental health service system to meet these challenges, problems remain, including long waits for care, low levels of uptake and engagement once in care, and modest treatment effects for those who overcome access and engagement challenges [4]. Youth mental health services, like all mental health services, face a sequential and compounding series of challenges that ultimately lead to low levels of overall effectiveness relative to community needs.

Fewer than half of all young people with mental health conditions seek or receive evidence-based treatment [5-7]. Among the key structural barriers to effective care is that the demand for care outstrips the supply of qualified mental health professionals, resulting in both excessive wait times and premature discharge. Young people also tend to disengage early from interventions. About 42% of young people drop out of treatment by the third therapy session in face-to-face service settings [8], and those with more complex mental health problems have poor attendance [9]*.* Even when young people receive evidence-based psychological interventions, their effectiveness is often limited, with effect sizes ranging from small to moderate [10]. A review of 453 randomized controlled trials over 50 years revealed that psychological treatment effectiveness has not improved for youth anxiety and has decreased for youth depression [11]. Furthermore, despite the exceedingly high rates of diagnostic comorbidity and symptom heterogeneity seen in youth mental health services, most evidence-based psychological interventions tend to be single-disorder-focused, one-size-fits-all approaches derived from trials reporting group-level effects from selected participants [12]. In care settings and clinical trials, comorbid conditions are typically ignored, and disorders are treated as a rigid constellation of symptoms [13]. This approach has left us with interventions that work well for the “average” young person with a narrow and specific set of presenting difficulties, leading clinicians and services to personalize interventions through a trial-and-error approach [14]. After more than 50 years of randomized controlled trials focused on group-effect research, we simply do not know what works, for whom, and why [15].

### The Role of Digital Technology in Youth Mental Health Care Reform

Digital technologies have the potential to enhance care access, engagement, and treatment effectiveness. Nearly 30 years of internet-delivered treatment research and service delivery for adults have demonstrated that these services significantly improve accessibility while producing engagement and effectiveness outcomes that are roughly equivalent to face-to-face psychological therapy [16-18]. Mental health system reviews have called for digital interventions to be “blended into service delivery,” which has now become a national and international policy priority [19,20]. The recent Australian Royal Commission into Victoria’s Mental Health System found that the mental health system is “antiquated” and “has failed to keep up to date with advances in digital technology.” It mandated a contemporary “system enabled by digital technology” to “improve accessibility and continuity of care” [21].

Much like face-to-face services, however, uptake, engagement, and adherence to digital interventions remain challenges [19]. The problem appears to be exacerbated in young people, where rates of engagement in established digital service offerings are lower than those in adults [18,22]. Additionally, there has been a historical, global failure to integrate digital interventions into real-world mental health services [19]. Innovative mental health solutions with matched implementation and integration plans are urgently required to effectively overcome these challenges.

### Moderated Online Social Therapy

Moderated Online Social Therapy (MOST) [23] is a digital mental health platform (both a web interface and a dedicated app) for people aged between 12 and 25 years, designed to be integrated (blended) into established face-to-face youth mental health services, including primary care mental health services like “Headspace” and specialist child and adolescent mental health services [4]. Mental health professionals and researchers can refer young people to the platform to use any of the five interacting components: (1) evidence-based psychotherapeutic content, including psychoeducation, reflective activities, behavioral experiments, and comics; (2) additional remote clinician support; (3) remote peer support; (4) remote vocational or career consultation support; and (5) a peer-led web-based community and social network. These distinct components are designed to work cohesively together, enabling young people and their treating Mental health professionals to choose the kinds of support that best suit their needs. For instance, some young people might only access therapeutic content, while others might only use vocational support. However, the majority of users access more than two or more components, depending on their preferences and needs. In this way, MOST is a flexible service offering that can be adapted to a range of different preferences, needs, and service delivery contexts.

**Figure 1:**
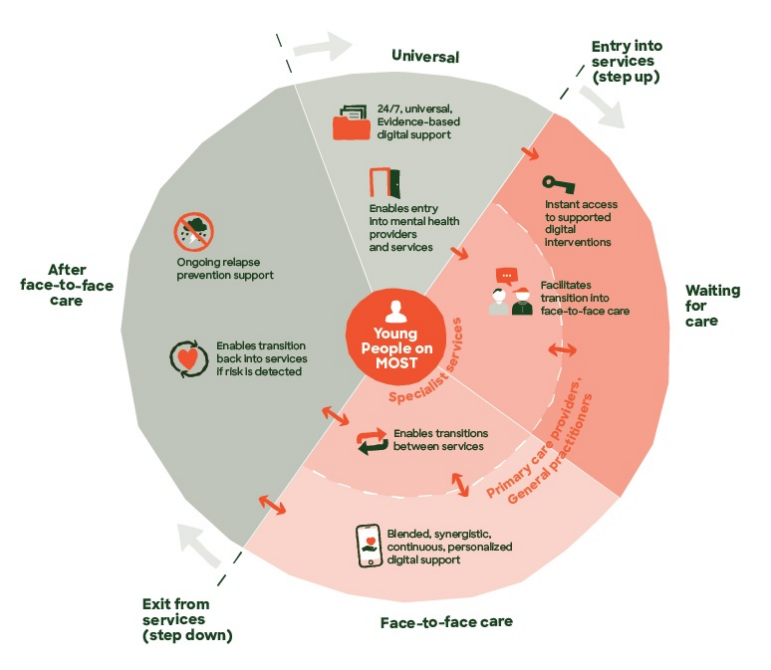
Moderated Online Social Therapy (MOST) use cases and intended purpose at different phases of treatment: from before treatment (universal), to waiting for care, to engaging in face-to-face care in services, to being recently discharged from care.

The flexibility of the platform allows MOST to be used differently by young people in youth mental health services at different phases in the care pathway (Figure 1). For instance, at the point of service entry, rather than waiting for a face-to-face mental health professional to be available, MOST can be used by the young person to gain immediate access to evidence-based care and remote professional and social support. At the point of receiving face-to-face care from a mental health professional, personalized clinical and psychosocial content can be recommended, homework can be scheduled, appealing strategies can be reflected on, and social or peer support can be provided between sessions. After care or at discharge, MOST can be used to maintain treatment gains, reduce relapse, or be an ongoing resource for treatment and support for those who drop out of care early. MOST can also be used for young people not connected to the formal care system at all, potentially alleviating demands on the mental health system and facilitating entry into mental health services as needed.

### Theory of Change

Improving access, engagement, and effectiveness of youth mental health services is a multifaceted endeavor that likely requires several interacting interventions. To explain how an intervention with multiple interacting components (known as a complex intervention) works, the Medical Research Council’s framework for the evaluation of complex interventions [24,25] recommends developing a theory of change (ToC) or a program logic. As a theory-driven evaluation technique, a ToC seeks to understand how and why a complex intervention works to guide evaluation and refinement [26]. Whereas a logic model is a description of the process of delivering an outcome through connecting inputs, activities, and outputs, a ToC overcomes a weakness of this approach by also articulating the assumptions and the theory or proposed explanations as to how the change process will occur [27]. A ToC entails a process of formulating how a complex intervention is supposed to work, why it works, who benefits and how, and what conditions are required for success. Through participatory techniques, a ToC produces pathways and quantifiable indicators that remain changeable as an intervention unfolds [28]. A ToC is often expressed visually in an accompanying narrative, showing the casual pathways and the evidence that links them, which can come from a variety of sources, including research evidence, behavior change theories, lived experience knowledge, or research conducted as part of the intervention feasibility and piloting stage [29].

## Methods

### Overview

MOST brings together several theoretical perspectives on behavior and therapeutic change in young people in the context of mental health challenges. Further, the ToC describes how a digital platform like MOST is designed to integrate within established face-to-face youth mental health services, thereby enhancing service provision and ultimately leading to a range of improved outcomes.

The MOST ToC provides a framework to evaluate the real-world impact of digitally enhancing a large nationwide network of youth mental health services and to hypothesize the therapeutic mechanisms and contextual factors that drive effectiveness in clinical settings, thus allowing iteration and refinement of the platform itself.

Over a 12-year period, more than 1000 consultation and co-design sessions have been conducted with young people, clinicians, and youth mental health services, and their findings have been reported within the various studies using the MOST platform as outlined below.

The development of the MOST ToC was conducted in 5 steps, as outlined below.

1. Findings from 2 completed and 4 ongoing randomized control trials and 11 pilot studies across 44 youth mental health services were reviewed. Over time, MOST has been customized to address several mental health conditions and associated challenges, including psychosis [30], ultra-high risk for psychosis [31], depression [32], anxiety [33,34], vocational recovery [35], and suicide risk [36], across all stages of treatment (ie, help-seeking, blended with face-to-face care, and relapse prevention). In summary, results from these trials demonstrate that MOST is (1) engaging, with 50% of young people with psychosis engaged over 9 months or longer and 70%-80% of young people across the diagnostic spectrum engaged for 3 months in shorter-term studies; (2) safe, with no incidents and serious adverse events over 11 years of testing; (3) endorsed by young people (95% of young people would recommend it to others) and clinicians (100% considered it helpful for young people); (4) effective in improving vocational recovery and reducing hospital admissions and visits to emergency services in young people with psychosis; (5) cost-effective and cost-saving [37]; and (6) promising in improving depression, anxiety, psychological distress, social functioning and support, loneliness, and well-being.
2. A review of qualitative findings from young people engaging in MOST in a variety of contexts was done; these included: MOST design and development with young people with first-episode psychosis [38]; MOST social networking use with active suicidal ideation [39]; social anxiety in psychosis [40]; social anxiety [41]; depression relapse prevention [42]; experience of intervention for first-episode psychosis [43,44]; and blended care experiences for first-episode psychosis [45].
3. A review of the recent digital mental health literature and evidence-based theories for guidance on improving digital engagement and adherence, improving outcomes for diagnostically diverse young people, and facilitating digital platform implementation in traditional services.
4. Examples of ToC frameworks in similar yet distinct youth web-based mental health programs [46] were undertaken, leading to the construction of a ToC framework (Textbox 1) that outlines the ToC components and describes how each element logically relates to the preceding and proceeding elements.
5. A final review and feedback from a range of MOST experts (study authors) who have variously been central in the development and service delivery of MOST over the last 12 years.

**A theory of change (ToC) framework outlining definitions of specific elements.**

**Overall mission and purpose**

- What is the overarching purpose and mission of the program?

**Values**

- What values guide the approach to the program?

**Assumptions**

- What key assumptions are made that will influence the program and its outcomes? Are there any implicit or widely accepted assumptions?

**Contextual factors**

- What is the external context in which this program sits? Which factors might impact the program and its outcomes?

**Inputs and resources**

- What resources are needed to meet the overarching purpose of the program?

**Participants and stakeholders**

- Who are the key stakeholders the program is trying to promote change with?

**Problem statement and stakeholder challenges**

- What problems or challenges do these stakeholders face that the program is trying to address directly or indirectly?

**Guiding theories and approaches**

- What are the theories that can guide changes to these problems or challenges?

**Mechanisms of change**

- What are the key change-inducing targets or mechanisms that are informed by these theories that guide the activities (interventions) that lead to the change (usually based on science, data, or internal experience)?

**Activities and interventions**

- What are the “key ingredients,” or the activities or interventions that promote the change? Are these guided by mechanisms or overarching theories?

**Outputs (observable)**

- What is the evidence that the activities or interventions were performed as planned (eg, the quantity of what is delivered)?

**Short-term (measurable) outcomes**

- What kind of short-term (eg, weeks) changes came about because of the program, be they intended or unintended?

**Medium-term outcomes and wider benefits**

- What kind of medium-term (eg, months) changes came about because of the program, be they intended or unintended? Are there wider benefits realized over time?

**Long-term outcomes and impact**

- At a larger scale over a longer period (eg, years), what are or could be the broader society-level impacts?

### Ethical Considerations

This study is a synthesis of data from published research, so no ethics review was required.

## Results

### Overview

The above steps led to the production of the first version of the MOST ToC, which is presented in both narrative form and in a more detailed format (Textbox 2). Figure 1 summarizes the use cases of MOST at various phases of the care pathway.

**Detailed summary of the MOST (Moderated Online Social Therapy) Theory of Change (ToC).**

**Overall mission and purpose**

- Integrate effective human professional support, be it remote or face-to-face, with digital health technology in order to personalize mental health care and services for young people.

**Assumptions**

- A proportion of young people in various stages of care (waiting, in face-to-face care, discharged, or referred) want or are able to use digital tools to manage their mental health and vocational needs.
- A proportion of clinicians, vocational workers, and peer workers want or are able to use digital tools to help them support young people.
- A proportion of services want or are able to be digitally enhanced or integrated, whereby digital tools are used to enhance all aspects of service quality (including accessibility, efficiency, effectiveness, and satisfaction).

**Contextual factors**

- Rates of mental illness in young people are very high, and traditional youth mental health services are not coping with service demand.
- The digital mental health landscape in Australia and internationally (both public, private, and nonprofit offerings) is expanding and difficult for users to navigate.
- Policy reviews support digital integration, though there is a lack of infrastructure for digital integration, and so digital offerings sit disconnected from public mental health offerings.
- Significant change is occurring in youth mental health services in light of current reform agendas.

**Inputs and resources**

- Workforce (clinicians, researchers, engineers, designers, content developers, peer support workers, career consultants, and implementation team).
- Funding and partnerships.

**Participants and stakeholders and their challenges and problem statement**

- Young people and MOST clinical, peer, and vocational staff.
  Lack of timely access to evidence-based intervention and support from services (access).
  Lack of social support, high levels of social isolation, a lack of sense of belonging, and a lack of normalization of experience.
  Lack of treatment and the right level of treatment intensity matched to both clinical needs and young people’s preferences (personalization).
  Poor treatment outcomes (low engagement and symptom or functional improvement; effectiveness).
  Lack of support and resource access between face-to-face sessions (care continuity and treatment intensity).
  Most clinical resources provided in care are paper-based, from a variety of sources, and difficult to coordinate and manage over the course of treatment.
  Young people exiting care still with clinical needs.
  Lack of aftercare maintenance and relapse prevention support (care continuity).
- Young people and their external clinicians and services
  Demand for services is higher than supply of clinicians and interventions (access).
  Lack of time to treat large numbers of young people (high demand) and lack of time to support young people between sessions (efficiency).
  Lack of feedback about what is working or not working for young people to guide therapy, clinical management, and clinical decision-making (personalization).
  Difficulty implementing gold standard treatments due to limited capacity (efficiency)
  Difficulty finding, vetting, and managing clinical resources that are mostly “paper-based.”
  Time-limited interventions.
  Difficulty discharging young people with improved outcomes to suitable services.
  Young people returning to care shortly after discharge (failure demand).

**Guiding theories and approaches**

- Self-determination theory (SDT) of motivation [47].
- Transdiagnostic, mechanistic, and process-based approaches to matched psychological interventions.
- Behavior change and motivational therapies
- RE-AIM (Reach, Effectiveness, Adoption, Implementation, and Maintenance) outcome framework [48].
- Adaptive, tailored, and behavioral science-informed (ATLAS) implementation framework (including the “Consolidated Framework for Implementation Research” [CFIR] [49], “Expert Recommendations for Implementing Change” [ERIC] [50], and “nonadoption, abandonment, scale-up, spread, and sustainability” [NASSS] [51]).

**Mechanisms of change—cross-cutting mechanisms**

- Platform design and content aim to promote the self-determined motivation of the young person, clinicians, and services to uptake and engage with platform content over time.
- Professional and peer support aims to promote therapeutic relationships and working alliances.
- Use of SDT-behavior change techniques [52] by support staff designed to enhance behavioral change.

**Mechanisms of change: component specific mechanisms**

- MOST therapeutic content and clinician support.
  Fidelity to the Clinical Support Model.
  Transdiagnostic mechanisms targeted by interventions (and clinically recommended) include, but are not limited to, repetitive negative thinking, cognitive and affective biases, experiential avoidance, and motional dysregulation.
- MOST peer support.
  Fidelity to the Peer Support Model.
- MOST social network.
  Fidelity to the Social Network Moderation Model.
- MOST career support.
  Fidelity to the Career Support Model and 8 principles of Individual Placement and Support (IPS) [53].
- MOST use by external clinicians (Blended Care)
  Fidelity to the Blended Care Model.
  Fidelity to ATLAS.
  Reduced complexity (measured by NASSS [51]).

**Activities (interventions)**

- MOST therapeutic content
  Evidence-based treatment options based on presenting difficulties.
  Personalized therapy toolkit.
  On-demand strategies.
- MOST clinician support.
  1:1 clinician support using clinical content.
  Safety management.
  Communications or referrals to other health professionals.
- MOST peer support.
  Moderated intentional peer support.
- MOST social network
  Moderated and safe web-based social networking.
- MOST career support.
  Tailored career content and journey.
  1:1 career consultant support.
- MOST use by external clinicians (Blended Care)
  MOST implementation team provides training and support to service-based clinical staff.
  Assistance to service to integrate MOST into clinical pathway.

**Outputs**

- Initial sign up and login into MOST.
- Completion or engagement in combinations of chosen journeys and completed therapeutic activities within the journeys.
- Interactions with a MOST clinician (number and time frame).
- Interactions with a MOST vocational worker (number and time frame).
- Interactions with a MOST peer worker (number and time frame).
- Interactions in the social network (number and time frame).
- Ongoing use (logins) of MOST.
- Clinician attendance at training.
- Clinician initial sign up to a MOST account.
- Clinician use of MOST and its features within face-to-face sessions.

**Short term (measurable) outcomes**

- Increased reach: proportion of young people onboarded to MOST.
- Reduced waiting time: time between service entry and treatment allocation.
- Reduced depression and anxiety symptoms.
- Improved functioning.
- Improved well-being.
- Improved vocational and educational attainment.
- High levels of young people’s satisfaction.
- High clinician acceptability.
- High clinician adoption: n (%) of clinicians using MOST.
- Fewer face-to-face sessions (Blended Care).
- Improved integration and normalization of MOST within the service’s routine and ongoing operations.

**Medium-term outcomes and wider benefits**

- Improved quality-adjusted life years (QALYs).
- Greater service throughput/numbers of young people serviced per annum.
- Lower rate of visits to emergency services and hospital admissions.

**Longer term outcomes and impact**

- Reduced annualized health care costs.
- Reduced societal costs of untreated disorder.
- Reduced burden of mental illness in young people and higher productivity.
- Improved accessibility, effectiveness, personalization (appropriateness) and efficiency of care across youth mental health services.

### Narrative Summary of the MOST Theory of Change

The mission of MOST is to integrate effective human professional support, be it remote or face-to-face, with digital health technology to personalize mental health care and services for young people. Technology-enhanced care centered around the needs and preferences of each young person can personalize mental health care.

There is growing interest in developing a new generation of integrated digital services in youth mental health services [4]. Various policy reviews report that the mental health system is fragile and not meeting the needs and preferences of the young people who rely on it [20,21,54]. When it comes to digital integration, these reviews share common findings: that the system is technologically antiquated and that the most obvious and impactful way to achieve true system reform is through the integrated use of digital technology [21]. Several assumptions exist, centered around the readiness and uptake of these integrated digital solutions by young people, clinicians, and service providers [55,56]. There is clearly a demand worldwide for digital mental health solutions [57]. However, young people have comparatively lower rates of uptake of such digital health tools compared to adults [18], and clinicians and service providers experience several barriers to digital adoption in their challenging service delivery environments [58]. These and other external factors include an unprecedented youth mental health need in the community [59], increasing competition in the digital mental health service landscape, with a focus on user engagement over outcomes [60], and a lack of infrastructure and change management for services and clinicians to integrate digital technologies into their clinical practice.

The challenges can be overcome with effective inputs and resources. They include a diverse and highly skilled workforce of clinicians, peer support workers, career consultants, implementation officers, software engineers, user experience and user interface designers, professional writers, artists, graphic designers, researchers, and operational specialists who work together to develop and maintain the service. MOST is delivered with financial support from various Australian state governments, as well as two major national foundations, numerous research grants, and partnership support from a number of national and international research partners.

Our key participants and stakeholders are young people, their families and caregivers, and the service clinicians and services that provide clinical care. Each of these participants faces a set of unique challenges and problems that MOST seeks to address. For young people, these include difficulties navigating the mental health system, a lack of timely access to mental health care and support [59], a dearth of other specialized supports such as peer and vocation support, and a lack of appropriate (matched to needs and preferences) [61], continuous [62], and effective care [11]. Multidisciplinary youth mental health teams, clinicians, and peer and vocational workers experience challenges related to managing large caseloads of young people with complex needs [63], keeping up with administrative and clinical demands, finding time to provide effective care between formal appointments, having effective tools to tailor therapy and track clinical and vocational outcomes [64], and limited integrated digital tools to support scarce service resources and clinical workflow management. As a result, long waiting times [63] and disengagement from care are common [8,9], and a lack of digital integration within and between services causes inefficiencies and additional challenges when implementing new technologies [58].

Theories and approaches guide both the implementation of MOST, that is, its integration within face-to-face services, and its therapeutic foundation. Implementation is guided by the “adaptive, tailored, and behavioral science-informed” (ATLAS) implementation strategy framework, bringing together gold-standard determinants such as Consolidated Framework for Implementation Research (CFIR) [49], Expert Recommendations for Implementing Change (ERIC) [50], and technology implementation (nonadoption, abandonment, scale-up, spread, and sustainability [NASSS]) frameworks [65]. Established theories and approaches to mental health support are evident in the literature, most notably self-determination theory (SDT) [66] and transdiagnostic, mechanistic, process-focused psychological therapy approaches [13,14,67,68]. Derived from these theories and emerging evidence, MOST targets key mechanisms of change, such as behavior change techniques used in the platform and by MOST clinicians, peer and vocational support to improve engagement [52], and transdiagnostic therapy targets, including but not limited to, repetitive negative thinking [69], cognitive and affective biases [70], experiential avoidance [71], and emotional dysregulation [72], to improve effectiveness. These mechanisms of change guide and inform the activities and interventions in MOST that include psychotherapeutic content, clinician support, peer support, a social network, vocational support, and implementation support, which are ultimately designed to interact and make an impact on the problems and challenges that bring a young person to the platform.

Measuring the outputs of these activities ensures that MOST is collecting and monitoring the kinds of data that link the activities with the problem targets. These outcomes are divided into short-, medium-, and long-term outcomes. While each of the activities is listed by component, they share common outputs and outcomes, such that unique combinations of these activities within MOST can achieve a diverse set of outcomes, from symptom reduction, social and functional recovery, and vocational attainment, to improved access, care continuity, service efficiency, and digital integration.

## Discussion

### Overview

As is common in ToC development, the creation of the first version of the MOST ToC helped us to connect the intended primary set of outcomes for the complex intervention, to articulate the inputs, activities, and outputs, and to make explicit the underlying assumptions and context of the intervention, as well as the theories and underlying mechanisms stakeholders believe will drive meaningful change. As a result, the ToC becomes a framework for ongoing evaluation and testing of MOST, helping identify areas for further development in MOST and iteratively improving the ToC itself, which we outline below.

### Evolving the Therapeutic Content to Better Target Transdiagnostic Mechanisms

The construction of the ToC highlighted the need to improve treatment outcomes for young people with a range of different mental health problems and disorders, not just high-prevalence single disorders. This broader approach may be achieved by better targeting “transdiagnostic mechanistic constructs” at the level of the individual [73]. Transdiagnostic approaches may [14,74] be more precise, personalized, easy to use, and can be used with large-scale implementation of complex interventions [13,75]. Multiple meta-analyses also indicate that, relative to diagnostic-specific interventions, transdiagnostic protocols are at least as or more effective at addressing the primary clinical diagnosis, more effective at alleviating comorbid diagnoses, easier to implement, and more engaging [47,76-78].

MOST’s therapeutic content is grouped into web-based guided transdiagnostic or problem-specific “journeys” that contain evidence-based techniques from 2nd- and 3rd-wave cognitive behavioral therapies (CBTs), including CBT [79], acceptance and commitment therapy (ACT) [80], dialectical behavior therapy (DBT) [81], and mindfulness-based cognitive therapy (MCT) [82]. Within these journeys are specific skills-based activities that will better target a range of mechanisms outlined above and are directly supported by MOST and service clinicians, essentially blending their remote or face-to-face care with specific digital therapy content.

Future development of therapeutic content informed by the ToC will also seek to match content to specific transdiagnostic mechanisms as well as present problems. Further, it aims to use a more personalized approach rather than a single diagnosis and a group-informed approach to intervention by targeting individually specific mechanisms or transdiagnostic processes to improve engagement and outcomes, as well as make the intervention more efficient and less burdensome. In a newly funded clinical trial, we will test the effectiveness of this model using a wider range of mechanisms and carefully examine their interactions with individual characteristics, problems, and clinical outcomes in an effort to enhance treatment precision at the individual level.

### Enhancing Engagement and Adherence: Developing Peer and Professional Support Alongside the Social Network

Contemporary behavioral science theories such as SDT are increasingly applied to web-based service contexts to enhance engagement and adherence in health behavior change efforts. SDT is the most prominent and empirically supported theory of human motivation that has demonstrated efficacy in predicting motivated behavior in multiple contexts and populations and for a variety of health behaviors such as physical activity, healthy eating, and smoking cessation [83]. It is a theory of human motivation that focuses on the quality of motivation, or the internal reasons that drive behavior. The quality of motivation is influenced by the extent to which individuals experience support for 3 basic psychological needs: autonomy, competence, and relatedness. Therefore, behaviors or messages from agents that support the satisfaction of these needs are likely to promote autonomous motivation and sustained behavior change within the individual, while those that do not may undermine autonomous motivation and lead to negative outcomes, such as disengagement or poor adherence. Teixeira et al [52] outlined 21 behavior change techniques drawn from SDT that can guide change agent behavior to promote sustained engagement in a health intervention, and these techniques shall guide peer, vocational, and clinician interactions with young people as well as the design of MOST, including the therapeutic content.

Aligned with the ToC, future improvements to professional and peer support as well as the social network will include alignment and fidelity to SDT and related motivation and behavior change techniques (MBCT) [52] to provide support that promotes engagement and adherence. Clinicians, peer workers, vocational consultants, and social network moderators will be trained in behavior change techniques, and newly developed fidelity measures will track compliance.

### Improving Clinician and Service Adoption Through Implementation Science Frameworks

MOST is intended for integration within traditional youth mental health services to help address the multiple challenges experienced by the broader system. For example, integration of MOST at intake or entry can provide young people with immediate support while they would otherwise be waiting for care, or at discharge planning, where young people have ongoing access to relapse prevention support and content. Importantly, MOST can be used by clinicians in services, working in partnership with young people to augment their shared therapy goals and tasks with specific and relevant clinical content on the platform and concurrent remote peer and vocational support. In this way, MOST is an example of blended care [64]. There is evidence that blended care can be as effective as traditional therapy while requiring fewer sessions [84], potentially making it more scalable. Blended care may also improve engagement and compliance by linking digital tools to the client’s goals and providing a human component for building a therapeutic alliance.

The implementation of MOST in face-to-face services will be guided by a purpose-built framework (ATLAS) that combines several highly researched and validated implementation approaches (Textbox 2). ATLAS will apply CFIR to identify barriers and facilitators to integrating MOST, match determinates to behavior change and contextual constructs, and select operationalized behavioral change strategies matched to ERIC implementation strategies. These steps result in a cohesive implementation intervention delivered to key stakeholders, including service leaders, clinicians, and administrative personnel, and adapted at all levels overtime to address arising needs. Guided by the Reach, Effectiveness, Adoption, Implementation, and Maintenance (RE-AIM) outcome framework [48], these groups have been selected based on the primary implementation outcomes (reach and adoption), with service leaders having to endorse MOST’s adoption and integration, clinicians having to refer young people and adopt MOST in clinical practice, and administrative staff having to integrate MOST into services’ routine processes.

Integrating MOST in established clinical service environments is a key outcome in the ToC. Enhancing clinician and service adoption and the blended use of MOST in standard clinical care, as well as identifying facilitators, barriers, and the value of MOST implementation, are key areas for further development.

### Strengths and Limitations

Using a ToC helps articulate the theories, mechanisms, and approaches drawn upon that are hypothesized to facilitate meaningful change. These hypotheses can be tested, allowing the ToC itself to iterate and be constantly aligned with the latest evidence of what is effective as well as discarding what is not effective in driving change. Despite being a time-intensive exercise, the development of a ToC has led to new areas of platform and service development that otherwise may not have been highlighted and has strengthened several other areas, including service quality, clinician fidelity, and the monitoring and evaluation approach. We argue that there is value in other digital services constructing their own ToC frameworks.

While these main strengths support the development of a ToC for MOST, there are also several limitations. First, there is no established or standardized methodology for developing a ToC. Further iterations of the ToC will also help determine the most effective and feasible way to consolidate the vast amounts of knowledge from stakeholders, data from the digital service, and the broader scientific literature. Second, while the value of codeveloping a ToC with stakeholders is self-evident, the costs and time to develop them may be prohibitive to many and also difficult to repeat regularly. Third, choosing between a myriad of theories and approaches for addressing common difficulties in youth mental health service provision may be subject to bias. The theories chosen here were based on those with the most substantial and compelling contemporary evidence that were most adaptable to the digital service model. In time, the data will ultimately support or refute the use of these theories, highlighting the importance of using a ToC framework to articulate how each step in the process will ultimately be evaluated.

### Conclusions

MOST has been developed and tested over the past 12 years. By using a ToC framework, key processes within the complex intervention have been articulated that allow for future studies using MOST to capture key data points in their evaluations of effectiveness. Other complex interventions, including other multicomponent digital intervention platforms, may also benefit from developing a ToC to clearly articulate how the intervention is supposed to work, why it works, who benefits and how, and what conditions are required for success.

## Appendix

Multimedia Appendix 1 Detailed summary of the Moderated Online Social Therapy Theory of Change.

## Abbreviations

ACT: acceptance and commitment therapy
ATLAS: adaptive, tailored, and behavioral science-informed
CBT: cognitive behavioral therapy
CFIR: Consolidated Framework for Implementation Research
DBT: dialectical behavior therapy
ERIC: Expert Recommendations for Implementing Change
MBCT: motivation and behavior change technique
MCT: mindfulness-based cognitive therapy
MOST: Moderated Online Social Therapy
NASSS: nonadoption, abandonment, scale-up, spread, and sustainability
RE-AIM: Reach, Effectiveness, Adoption, Implementation, and Maintenance
SDT: self-determination theory
ToC: theory of change
