# Supplementary material for: Developing a Theory of Change for a Digital Youth Mental Health Service (Moderated Online Social Therapy): Mixed Methods Knowledge Synthesis Study
Source: JMIR Form Res. 2023 Nov 3;7:e49846. doi: 10.2196/49846 (PMC10656668; doi:10.2196/49846)
Supplement: Multimedia Appendix 1 [file formative_v7i1e49846_app1.docx]

**Table S1**. Detailed summary of the Moderated Online Social Therapy Theory of Change.

| **Overall Mission/ Purpose** | | - Integrate effective human professional support, be it remote or face-to-face, with digital health technology, in order to personalize mental health care and services for young people. | | | | | |
| --- | --- | --- | --- | --- | --- | --- | --- |
| **Assumptions** | | - A proportion of YP in various stages of care (waiting, in face-to-face care, discharged/referred) want/ are able to use digital tools to manage their mental health and vocational needs. - A proportion of clinicians, IPS vocational workers and peer workers want/ are able to use digital tools to help them support YP. - A proportion of services want/are able to be digitally enhanced/integrated, whereby digital tools are used to enhance all aspects of service quality (including accessibility, efficiency, effectiveness, satisfaction). | | | | | |
| **Contextual Factors** | | - Rates of mental illness in YP are very high, and traditional YMHSs are not coping with service demand. - The digital mental health landscape in Australia and internationally (both public, private, and non-profit offerings) is expanding and difficult for users to navigate. - Policy reviews support digital integration, though there is a lack of infrastructure for digital integration and so digital offerings sit disconnected from public mental health offerings. - Significant change is occurring in YMH services in light of current reform agendas. | | | | | |
| **Inputs / Resources** | | Workforce (Clinicians, Researchers, Engineers, Designers, Content Developers, Peer Support Workers, Career Consultants, Implementation Team), Funding, Partnerships. | | | | | |
| **Participants / Stakeholders** | | Young People and MOST Clinical, Peer and Vocational staff. | | | | | Young People and their External Clinicians/ Services. |
| **Stakeholder Challenges / Problem Statement** | | - Lack of timely access to evidence-based intervention and support from services (Access). - Lack of social support, high levels of social isolation, lack of sense of belonging, lack of normalisation of experience. - Lack of treatment and right level of treatment intensity matched to both clinical needs and YP preferences (Personalisation). - Poor treatment outcomes (1. Low engagement and 2. Symptom/ functional improvement) (Effectiveness). - Lack of support and resource access between face-to-face sessions (Care Continuity and Treatment Intensity). - Most clinical resources provided in care are paper based, from a variety of sources, difficult to coordinate and manage over course of treatment. - Young people exiting care still with clinical needs. - Lack of aftercare maintenance/ relapse prevention support (Care Continuity). | | | | | - Demand for services higher than supply of clinicians and interventions (Access). - Lack of time to treat numbers of YP (high demand) and lack of time to support YP between sessions (Efficiency). - Lack of feedback about what is working/ not working for YP to guide therapy, clinical management and clinical decision making (Personalisation). - Difficulty implementing gold standard treatments due to limited capacity (Efficiency) - Difficulty finding, vetting, and managing clinical resources that are mostly ‘paper-based’. - Time limited interventions. - Difficulty discharging YP with improved outcomes to suitable services. - YP returning to care shortly after discharge (Failure Demand). |
| **Guiding Theories and Approaches** | | - Self Determination Theory of Motivation (SDT). - Transdiagnostic, mechanistic and process-based approaches to matched psychological interventions. | | | | | - Behaviour change and motivational therapies - RE-AIM outcome framework. - ATLAS Implementation Framework (including CFIR, ERIC, and NASSS). |
| **Mechanisms of Change** | ***Cross Cutting Mechanisms*** | - Platform design and content aims to promote self-determined motivation of the young person, clinicians, and services to uptake, engage with platform content over time. - Professional and peer support aims to promote the Therapeutic Relationship and Working Alliance. - Use of SDT-BCTs by support staff designed to enhance behavioural change. | | | | | |
|  | ***Component Specific Mechanisms*** | Fidelity to Clinical Support Model.  Transdiagnostic Mechanisms targeted by Interventions (and clinical recommended) including but not limited to:   - Repetitive negative thinking. - Cognitive and affective biases. - Experiential avoidance. - Emotional dysregulation. | | Fidelity to Peer Support Model. | Fidelity to Social Network Moderation Model. | Fidelity to Career Support Model  8 principles of Individual Placement and Support. | - Fidelity to Blended Care Model. - Fidelity to ATLAS - Reduced complexity (measured by NASSS). |
| **Activities (Interventions)** | | ***MOST Therapeutic Content***  Evidence-based treatment options based on presenting difficulties.  Personalised therapy toolkit.  On-demand strategies. | ***MOST Clinician Support***  1:1 Clinician Support using clinical content.  Safety Management.  Communications/ referrals to other health professionals. | ***MOST Peer Support***  Moderated Intentional Peer Support. | ***MOST Social Network***  Moderated/Safe online social networking. | ***MOST Career Support***  Tailored career content / journey.  1:1 career consultant support. | ***MOST use by External Clinicians (Blended Care)***  MOST Implementation Team provides training and support to service-based clinical staff.  Assistance to service to integrate MOST into clinical pathway. |
| **Outputs** | | - Initial sign up and login into MOST. - Completion or engagement in combinations of chosen journeys and completed therapeutic activities within the journeys. - Interactions with a MOST Clinician (number and timeframe). - Interactions with a MOST Vocational Worker (number and timeframe). - Interactions with a MOST Peer Worker (number and timeframe). - Interactions in the Social Network (number and timeframe). - Ongoing use (logins) of MOST. | | | | | - Attendance at training. - Initial sign up to a MOST account. - Use of MOST and its features within face-to-face sessions. |
| **Short-term (measurable) outcomes** | | - Increased reach – proportion of YP onboarded to MOST. - Reduced waiting time – time between service entry and treatment allocation. - Reduced depression and anxiety symptoms as measured by PHQ9 and GAD7. - Improved functioning as measured by WSAS. - Improved wellbeing as measured by WEMWBS. - Improved vocational and educational attainment as measured by proportion working or enrolling in education (reduced NEET). - High levels of YP satisfaction. | | | | | - High clinician acceptability. - High clinician adoption – no. (%) clinicians using MOST. - Fewer face-to-face sessions (Blended Care). - Improved integration/ normalisation of MOST within service’s routine and ongoing operations (measured by NoMAD). |
| **Medium-term outcomes/ wider benefits** | | - Improved quality adjusted life years (QALYs) as measured by CHU9D. - Greater service throughput / numbers of YP serviced per annum. - Lower rate of visits to emergency services and hospital admissions. | | | | | |
| **Long-term outcomes and Impact** | | - Reduced annualised health care costs. - Reduced societal costs of untreated disorder. - Reduced burden of mental illness in young people and higher YP productivity. - Improved accessibility, effectiveness, personalisation (appropriateness) and efficiency of care across YMHSs. | | | | | |

SDT-BCT: Self Determination Theory Behavior Change Techniques [64]; PHQ9: Patient Health Questionnaire [69]; GAD7: Generalized Anxiety Disorder Questionnaire [70]; WSAS: Work and Social Adjustment Scale [71]; WEMWBS: Warwick-Edinburgh Mental Wellbeing Scale [72]; NEET: Not in education, employment or training; CHU9D: Child Health Utility instrument [73]; NoMAD: [74]; CFIR: the Consolidated Framework for Implementation Research [58], ERIC: Expert Recommendations for Implementing Change [59], NASSS Implementation Framework: [75]; ATLAS: Adaptive, TaiLored, and behAvioural Science-informed Implementation Strategy.
